# Assessing Patient Characteristics in Neuroendocrine Tumor Research: A Comparison of the NET-PRO Study to SEER Population-Based Data

**DOI:** 10.1007/s12020-026-04611-w

**Published:** 2026-04-27

**Authors:** Michael A. O’Rorke, Bradley D. McDowell, Tao Xu, Rhonda R. DeCook, Brian M. Gryzlak, Nicholas J. Rudzianski, Kimberly C. Serrano, Abigayle M. Wehrheim, Udhayvir S. Grewal, Chandrikha Chandrasekharan, Joseph S. Dillon, Thorvardur R. Halfdanarson, Michael J. Schnell, Carrie L. Witter, T. Clark Gamblin, Syed Kazmi, Lindsay G. Cowell, Tobias Else, Heloisa P. Soares, Vineeth Sukrithan, Sravani Chandaka, Hanna K. Sanoff, Fiona C. He, David A. Geller, Robert A. Ramirez, Mei Liu, William Lancaster, Josh A. Mailman, Heather Moran, Maryann Wahmann, Elyse Gellerman, Elizabeth A. Chrischilles

**Affiliations:** 1 College of Public Health, Department of Epidemiology, University of Iowa, 145 N Riverside Drive, Iowa City, IA 52242 USA; 2 Department of Internal Medicine, Carver College of Medicine, University of Iowa, Iowa City, IA USA; 3 Holden Comprehensive Cancer Center, University of Iowa, Iowa City, IA USA; 4 Department of Gastrointestinal Medical Oncology, The University of Texas MD Anderson Cancer Center, Houston, TX USA; 5 Mayo Clinic, Rochester, MN USA; 6 Medical College of Wisconsin, Milwaukee, WI USA; 7 Department of Internal Medicine, UT Southwestern Medical Center, Dallas, TX USA; 8 Department of Health Data Science and Biostatistics, O’Donnell School of Public Health, UT Southwestern Medical Center, Dallas, TX USA; 9 Department of Internal Medicine, Division of Metabolism, Endocrinology and Diabetes, University of Michigan, Ann Arbor, MI USA; 10 Huntsman Cancer Institute, University of Utah, Salt Lake City, UT USA; 11 Division of Medical Oncology, Department of Medicine, The Ohio State University Comprehensive Cancer Center, Columbus, OH USA; 12 University of Kansas Medical Center, Kansas City, KS USA; 13 Division of Oncology, Department of Medicine, University of North Carolina at Chapel Hill, Chapel Hill, NC USA; 14 Allina Health Cancer Institute, Minneapolis, MN USA; 15 University of Pittsburgh, Pittsburgh, PA USA; 16 Department of Internal Medicine, Division of Hematology/Oncology, Vanderbilt University Medical Center, Nashville, TN USA; 17 Department of Health Outcomes and Biomedical Informatics, College of Medicine, University of Florida, Gainesville, FL USA; 18 Department of Surgery, Medical University of South Carolina, Charleston, SC USA; 19 NorCal CarciNET Community, Oakland, CA USA; 20 The Healing NET Foundation, Los Angeles, CA USA; 21 Neuroendocrine Cancer Awareness Network, Fort Mill, SC USA; 22 Neuroendocrine Tumor Research Foundation, Boston, MA USA

**Keywords:** Neuroendocrine tumors, Patient-reported outcomes, SEER Program, Cohort studies, Real-world evidence

## Abstract

**Purpose:**

We compared demographic and clinical characteristics of patients with gastroenteropancreatic (GEP) and lung neuroendocrine tumors (NETs) enrolled in the NET-PRO study to those in the U.S. Surveillance Epidemiology and End Results (SEER) program to evaluate the comparability of NET-PRO as a resource for real-world evidence generation and patient reported outcomes (PROs).

**Methods:**

NET-PRO enrolled adults with GEP or lung NETs from 14 health systems between 2018 and 2024. SEER data included patients diagnosed with NETs from 2018 to 2021 across 22 U.S. cancer registries. We compared age, sex, race/ethnicity, tumor site, stage, and surgery between cohorts using standardized mean differences (SMDs), with values ≥ 0.2 interpreted as meaningful.

**Results:**

We analyzed 1,974 GEP-NET and 394 lung NET patients in NET-PRO versus 38,942 and 11,265, respectively, in SEER. Most demographic and clinical characteristics were broadly similar between cohorts, with trivial differences by sex (SMDs 0.16–0.22) and moderate differences in mean age (SMD = 0.47 for lung NETs). Race/ethnicity differences were larger, with Non-Hispanic White patients overrepresented in NET-PRO (SMDs 0.53–0.84). Tumor site and stage distributions differed modestly. Surgery rates were comparable for GEP-NETs but higher among NET-PRO lung NET patients (SMD = 0.47).

**Conclusion:**

NET-PRO demonstrates broadly comparable demographic characteristics to SEER across factors such as age and sex. While race/ethnicity differences highlight areas for improved inclusion, other areas of variation suggest important future research covariates. These findings support the contextual comparability of NET-PRO with the broader NET population and its value as a resource for real-world evidence generation.

**NCT Number:**

NCT05064150 (Start Date: 2022-05-10).

## Introduction

Neuroendocrine tumors (NETs) are uncommon, heterogeneous neoplasms arising most commonly in the small intestine, pancreas, and lung. Although relatively rare, their reported incidence has increased substantially over time [1], largely attributed to improvements in diagnostic imaging and clinical awareness rather than true changes in disease occurrence [2]. NETs often are associated with substantial symptom burden, and many patients experience diagnostic delays. Metastatic disease is common at presentation [3], and a subset, particularly those with small intestinal NETs, develop carcinoid syndrome [4]. Contemporary international guidelines from ENETS, NANETS, NCCN, and ESMO emphasize site-specific, risk-adapted management guided primarily by tumor differentiation and grade (e.g., Ki-67), stage/extent of disease, functional (hormone-secreting) status and somatostatin receptor imaging, together with patient factors such as performance status and comorbidity, underscoring the biological and clinical heterogeneity of NETs. As highlighted in recent precision-medicine frameworks for gastroenteropancreatic (GEP) NETs, few validated molecular predictors currently exist for NETs, and most treatment decisions remain grounded in multidisciplinary clinical assessment rather than established genomic targets; though theragnostic strategies and emerging actionable biomarkers show promise [5].

Despite these advances, important evidence gaps remain regarding treatment sequencing, long-term toxicities, and patient-centered outcomes. Because NETs often follow a prolonged clinical course, patients may receive multiple lines of therapy over many years, making symptom burden [6], treatment related effects, and quality of life central to shared decision-making. However, outside of clinical trials, real-world, patient-reported outcomes (PROs) data remains limited.

The Neuroendocrine Tumors–Patient Reported Outcomes (NET-PRO) Study was developed to address these gaps. NET-PRO is a large, multicenter, prospective cohort funded by the Patient-Centered Outcomes Research Institute (PCORI) that integrates longitudinal PROs with electronic medical record (EMR) data, including symptom burden, care experiences, treatment patterns, and health-related quality of life (HRQoL) among individuals with GEP and lung NETs across 14 health systems participating in the National Patient Centered Clinical Research Network (PCORnet^®^) [7]. Because NET-PRO is designed to inform comparative and patient-centered effectiveness research in a rapidly evolving therapeutic landscape, understanding how its participant characteristics compare with those of the broader U.S. NET population is important for interpreting the transportability of findings and identifying circumstances in which covariate adjustment may be required.

Few studies have systematically compared participants in large prospective NET cohorts with population-based registries to quantify potential differences in demographic and clinical characteristics. However, such comparisons are critical for interpreting findings from prospective cohorts intended to inform patient-centered and comparative effectiveness research. The Surveillance, Epidemiology, and End Results (SEER) Program provides high-quality, population-based cancer incidence data from 22 registries covering nearly half of the U.S. population [8]. In this study, we evaluated the demographic and clinical representativeness of NET-PRO participants by comparing our cohort characteristics with population-based cases from SEER.

## Methods

### NET-PRO Cohort

Eligible NET-PRO participants were adults (≥ 18 years) diagnosed with a GEP or lung NET between January 1, 2018, and September 30, 2024, and receiving care at one of 14 academic or community-based PCORnet health systems. Potentially eligible individuals were identified using a validated computable phenotype largely based on ICD diagnosis codes applied to EMR data, publicly deposited at PheKB. Patients were recruited using flexible, low-touch methods (e-mail, mail, phone, or in-clinic) and were enrolled and consented primarily (84.6%) through a personalized health record portal [9]. Further details on NET-PRO identification and enrollment are reported elsewhere [10]. A total of 2,538 patients enrolled; after excluding individuals who did not meet diagnosis timing criteria (*n* = 47), did not report a tumor location (*n* = 29), or reported both GEP and lung NETs (*n* = 94), the analytic sample included 2,368 participants.

### SEER Cohort

The comparison cohort was derived from the 2023 SEER Research Plus release, which includes data from 22 registries that cover almost 46% of the U.S. population [11]. We identified adults (≥ 18 years) diagnosed from 2018 to 2021 with malignant NETs of the lung or GEP tract. NETs were defined using ICD-O-3 histology codes (8150, 8151, 8152, 8153, 8155, 8156, 8240, 8241, 8242, 8246, 8249) and topography codes corresponding to lung (C34.X) and GEP sites (C16.X, C17.X, C18.X, C19.X, C20.X, C25.X, C26.X). For individuals with multiple records, the first eligible diagnosis was selected. Cases identified solely through autopsy or death certificate were excluded.

### Study Variables and Statistical Analysis

We extracted age at diagnosis, sex, race/ethnicity, primary tumor site, stage at diagnosis, and receipt of cancer-directed surgery. NET-PRO variables (including race/ethnicity) in NET-PRO were self-reported by participants during survey enrollment, whereas SEER variables are derived from medical record abstraction. Descriptive statistics were computed using R (R Foundation, Vienna, Austria) and SAS 9.4 (SAS Institute, Cary, NC, USA). Categorical variables are reported as frequencies and percentages; continuous variables as means with standard deviations (SD). Standardized mean differences (SMDs) were used to assess differences between NET-PRO and SEER; values of 0.2, 0.5, and 0.8 are commonly interpreted as small, medium, and large effect sizes, respectively. SMDs are widely used benchmarks for evaluating comparability between groups in observational research [12, 13]. SMDs were computed as the difference in means divided by the pooled standard deviation for continuous variables. For categorical variables, SMDs were calculated based on differences in proportions across groups. Two SMDs are provided for age (one for continuous age, one across categories). For race/ethnicity, ‘other’ was not available in SEER registry data, and so was not included as a category when calculating the SMD for this variable.

## Results

**Fig. 1:**
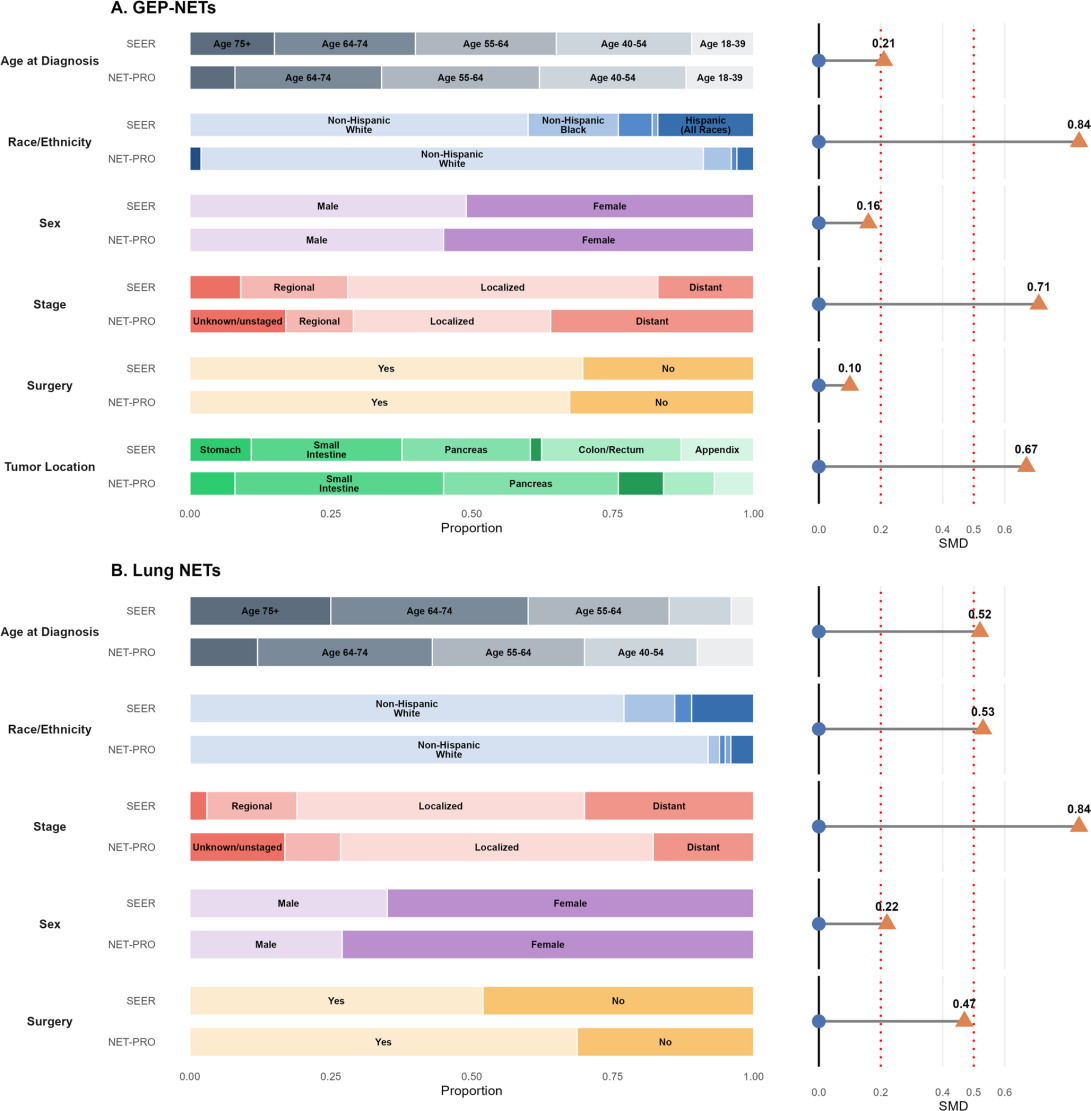
Demographic and clinical characteristics between the NET-PRO and SEER cohorts with standardized mean differences.

To evaluate representativeness of the NET-PRO cohort within the broader NET population, we compared demographic and clinical characteristics with SEER using SMDs. The SEER cohort included 38,942 individuals with GEP-NETs and 11,265 with lung NETs, compared with 1,974 GEP-NET and 394 lung NET participants in NET-PRO (Table 1; Fig. 1). Sex distributions showed small differences between cohorts (SMDs 0.16–0.22), with slightly more women represented in NET-PRO. NET-PRO participants were also modestly younger than SEER patients, particularly for lung NETs (mean difference ~ 6 years; SMD 0.47). Racial and ethnic diversity differed substantially. Non-White and Hispanic patients were underrepresented in NET-PRO relative to SEER across both tumor groups (SMDs 0.53–0.84), representing the largest observed demographic divergence.

**Table 1:** Comparison of demographic and clinical characteristics between NET-PRO enrollees to patients diagnosed with GEP or Lung NETs during 2018–2021 in 22 SEER Registries

	GEP-NETs				Lung NETs			
	SEER	NET-PRO	SMD*	Pvalue	SEER	NET-PRO	SMD*	Pvalue
	38,942	1,974			11,265	394		
**Sex**			0.16	< 0.001			0.22	0.003
Female	19,829 (50.9%)	1,075 (54.8%)			7,357 (65.3%)	283 (72.6%)		
Male	19,113 (49.1%)	887 (45.2%)			3,908 (34.7%)	107 (27.4%)		
**Race/Ethnicity**			0.84	< 0.001			0.53	< 0.001
Non-Hispanic White	22,956 (60.1%)	1,737 (89.7%)			8,637 (77.1%)	352 (90.7%)		
Non-Hispanic Black	6,182 (16.2%)	89 (4.6%)			1,017 (9.1%)	7 (1.8%)		
Non-Hispanic American Indian/Alaska Native	220 (0.6%)	7 (0.4%)			44 (0.4%)	3 (0.8%)		
Non-Hispanic Asian or Pacific Islander	2,355 (6.2%)	18 (0.9%)			320 (2.9%)	3 (0.8%)		
Hispanic (All Races)	6,474 (17.0%)	54 (2.8%)			1,190 (10.6%)	14 (3.6%)		
Other (checked multiple races)	n/a	31 (1.6%)			n/a	9 (2.3%)		
**Age at diagnosis (years)**,** mean (SD)**	59.2 (14.9)	57.4 (13.9)	0.13	< 0.001	65.8 (12.3)	59.7 (13.7)	0.47	< 0.001
18–39	4,298 (11.0%)	232 (11.9%)	0.21	< 0.001	449 (4.0%)	38 (9.8%)	0.52	< 0.001
40–54	9,392 (24.1%)	506 (25.9%)			1,252 (11.1%)	78 (20.1%)		
55–64	9,751 (25.0%)	544 (27.8%)			2,803 (24.9%)	106 (27.3%)		
65–74	9,595 (24.6%)	508 (26.0%)			3,943 (35.0%)	120 (30.9%)		
75+	5,906 (15.2%)	165 (8.4%)			2,818 (25.0%)	46 (11.9%)		
**Stage at Dx**			0.71	< 0.001			0.84	< 0.001
Localized	21,515 (55.3%)	700 (35.5%)			5,777 (51.3%)	219 (55.6%)		
Regional	7,328 (18.8%)	233 (11.8%)			1,787 (15.9%)	38 (9.6%)		
Distant	6,765 (17.4%)	711 (36.0%)			3,371 (29.9%)	70 (17.8%)		
Unknown/unstaged	3,325 (8.5%)	330 (16.7%)			329 (2.9%)	67 (17.0%)		
**Surgery at primary site**			0.10	0.005			0.47	< 0.001
Yes	26,859 (69.0%)	1,301 (65.9%)			5,806 (51.5%)	269 (68.3%)		
No	11,530 (29.6%)	634 (32.1%)			5,358 (47.6%)	124 (31.5%)		
Missing	553 (1.4%)	39 (2.0%)			101 (0.9%)	1 (0.3%)		
**Tumor location** **(GEP sites only)**			0.67	< 0.001				
Appendix	5,174 (13.3%)	122 (6.9%)						
Colon/Rectum (not including appendix)	9,556 (24.5%)	161 (9.1%)						
Pancreas	8,827 (22.7%)	552 (31.3%)						
Small Intestine	10,365 (26.6%)	658 (37.3%)						
Stomach	4,285 (11.0%)	134 (7.6%)						
Other and ill-defined digestive organs	735 (1.9%)	137 (7.8%)						

Abbreviations: SMD = Standardized Mean Difference, GEP = Gastroenteropancreatic, NET = Neuroendocrine tumor, SEER= Surveillance Epidemiology and End Results, NET-PRO = Neuroendocrine Tumors - Patient Reported Outcomes

*SMDs were computed as the difference in means divided by the pooled standard deviation for continuous variables. For categorical variables, SMDs were calculated based on differences in proportions across groups. Two SMDs are provided for age (one for continuous age, one across categories). For Race/Ethnicity, ‘other’ was not available in SEER registry data, and so was not included as a category when calculating the SMD for this variable.

Stage at diagnosis demonstrated marked imbalance. Among GEP-NETs, NET-PRO included fewer localized-stage and more distant-stage cases compared with SEER (SMD 0.71). For participants with lung NETs, stage distributions also differed substantially (SMD 0.84), with NET-PRO including a higher proportion of unknown stage and differences in the relative distribution of localized and distant disease.

Primary tumor distribution varied among GEP-NETs, with overrepresentation of small intestine (37.3% vs. 26.6%) and pancreatic NETs (31.3% vs. 22.7%) and underrepresentation of appendiceal, gastric, colonic, and rectal NETs (SMD 0.67). Surgical treatment rates were similar for GEP-NETs (SMD 0.10) but higher among NET-PRO lung NET participants (SMD 0.47). Collectively, the largest differences between cohorts were observed for race/ethnicity, stage, and GEP primary tumor site.

## Discussion

To our knowledge, few prior studies have systematically evaluated the representativeness of a large, multicenter, prospective NET cohort using population-based registry comparisons. Using SMDs, we found that most demographic characteristics of NET-PRO participants were broadly comparable with SEER, with small differences for sex and modest differences for age. Larger differences were observed for race/ethnicity and stage and primary tumor distribution, indicating areas where the NET-PRO cohort differs from the underlying U.S. NET population. Interpreting these differences relative to established SMD benchmarks helps contextualize the generalizability of NET-PRO for future PRO research, and whether findings can reasonably be interpreted within the broader real-world NET population.

Women were moderately overrepresented in NET-PRO compared to SEER, which may reflect the acceptability of NET-PRO’s pragmatic enrollment strategies [14] and the recognized higher incidence of lung NETs among women [15]. This contrasts with clinical trials, where women remain under-represented [16]. NET-PRO also achieved strong enrollment of younger lung NET patients; a group often underrepresented in clinical trials [17–19]. Although adults aged ≥ 75 years were less represented than in SEER, meaningful numbers were included, supporting analyses across age groups.

Race and ethnicity differences were more substantial. Non-Hispanic White participants were markedly overrepresented in NET-PRO relative to SEER for both GEP and lung NETs. These differences likely reflect variation in the demographic composition of participating health systems, language limitations of study materials, and differences in outreach capacity. Because racial and ethnic inequities affect diagnosis, treatment, and outcomes in NETs, future expansions of NET-PRO and similar initiatives should prioritize multilingual materials and partnerships with health systems serving more diverse populations to improve external validity. Beyond recruitment considerations, this imbalance has implications for health equity, as underrepresentation of racially and ethnically minoritized populations may limit the extent to which PRO findings reflect the experiences of all populations affected by NETs.

Differences in stage distribution between NET-PRO and SEER likely reflect referral patterns to tertiary centers, where NET-PRO sites are concentrated. For GEP-NETs, NET-PRO overrepresented distant-stage disease relative to SEER, consistent with more complex cases being managed at high-volume specialty centers. For lung NETs, NET-PRO included fewer distant-stage diagnoses, perhaps reflecting differences in referral, diagnostic workup, or surveillance practices. Interpretation of stage differences should also consider measurement non-equivalence between data sources. Stage in NET-PRO was self-reported by participants, whereas SEER stage is derived from clinical abstraction. However, preliminary validation among participants with completed chart abstraction demonstrates moderate agreement for stage (weighted Cohen’s kappa = 0.49, 95% CI [0.43, 0.55]), suggesting that the higher proportion of “unknown” stage in NET-PRO likely reflects incomplete patient knowledge of staging rather than true clinical differences.

Patient perceptions may also explain differences between NET-PRO and SEER regarding report of surgery: for lung NETs, 68.3% of NET-PRO patients report having cancer-directed surgery (yes response to “Excluding biopsies, have you received surgery to remove all or part of a neuroendocrine tumor?”), whereas only 51.5% of the SEER cohort had surgery documented in the medical record. While this may reflect true differences between the cohorts in terms of access to specialty care, or selection of surgical candidates at participating institutions, it could also be that lung NET patients considered procedures like bronchoscopy to be cancer-directed surgery. GEP-NETs comprise several tumor types, and there are many differences in proportion of these types between NET-PRO and SEER. NET-PRO overrepresented small intestine (37.3%) and pancreatic NETs (31.3%), and underrepresented appendiceal (6.9%) and colon/rectal NETs (9.1%), which may have implications for subtype-specific analyses. This distribution mirrors patterns seen in many specialty NET programs, where patients with small intestinal and pancreatic NETs are more likely to require multidisciplinary management and longitudinal specialty follow-up. In contrast, given that most appendix and rectal tumors are small, they may have little need for specialty consultation after appendectomy or rectal polyp removal. Despite these differences, surgery indicators for GEP-NET patients were more similar across NET-PRO (65.9%) and SEER (69.0%), suggesting more comparable treatment access or practice patterns for this subgroup. Lung NETs are relatively uncommon and generally receive less focused attention than GEP-NETs, yet NET-PRO enrolled 394 participants, representing 17% of the cohort and yielding one of the larger U.S. lung NET PRO datasets [20]. This supports the feasibility of prospective, multisite lung NET research. Finally, the descriptive and univariate nature of these comparisons does not account for potential interrelationships among demographic and clinical factors such as age, race/ethnicity, and tumor site. Future analyses using the NET-PRO cohort will incorporate multivariable adjustment to address these interdependencies.

NET-PRO has several strengths, including its prospective design, integration of PROs with EMR data, and participation of multiple academic and community health systems. However, several limitations should be considered. First, clinical characteristics in NET-PRO were self-reported, whereas SEER variables are abstracted from medical records, which may introduce misclassification for variables such as stage or surgery. EMR linkage will further validate and enrich these variables in future analyses. Second, because many NET-PRO sites are tertiary referral centers, the cohort may overrepresent patients with more complex disease, as reflected by the higher proportion of distant-stage GEP-NETs compared with SEER. Third, racial and ethnic diversity was lower in NET-PRO, which may limit generalizability and highlights the need for expanded recruitment strategies in future cohort expansions. Finally, differences in tumor site distribution likely reflect referral patterns and the clinical pathways through which certain NET subtypes are managed.

In summary, NET-PRO captures a broad spectrum of demographic and clinical characteristics that approximate those reported in SEER for many domains, supporting the cohort’s representativeness for real-world NET research. Areas of difference, including race/ethnicity, stage distribution, and primary tumor site, highlight important covariates that should be considered in future analyses and underscore opportunities to enhance recruitment diversity. Overall, NET-PRO constitutes a robust resource for generating patient-centered, real-world evidence to inform NET care and outcomes.

## Data Availability

The datasets generated and/or variables analyzed during the current study for consented patients (including questionnaires and data dictionaries) will be deposited to the Patient-Centered Outcomes Data Repository (PCODR) at the Inter-university Consortium for Political and Social Research (ICPSR) at the end of the study (via a de-identified data set).
